# mTORC Inhibitors as Broad-Spectrum Therapeutics for Age-Related Diseases

**DOI:** 10.3390/ijms19082325

**Published:** 2018-08-08

**Authors:** Hannah E. Walters, Lynne S. Cox

**Affiliations:** Department of Biochemistry, University of Oxford, South Parks Road, Oxford OX1 3QU, UK; hannah.walters@trinity.ox.ac.uk

**Keywords:** mTOR, mTORC1, mTORC2, rapamycin, rapalogues, rapalogs, mTOR inhibitors, senescence, ageing, aging, cancer, neurodegeneration, immunosenescence, senolytics, biomarkers

## Abstract

Chronological age represents the greatest risk factor for many life-threatening diseases, including neurodegeneration, cancer, and cardiovascular disease; ageing also increases susceptibility to infectious disease. Current efforts to tackle individual diseases may have little impact on the overall healthspan of older individuals, who would still be vulnerable to other age-related pathologies. However, recent progress in ageing research has highlighted the accumulation of senescent cells with chronological age as a probable underlying cause of pathological ageing. Cellular senescence is an essentially irreversible proliferation arrest mechanism that has important roles in development, wound healing, and preventing cancer, but it may limit tissue function and cause widespread inflammation with age. The serine/threonine kinase mTOR (mechanistic target of rapamycin) is a regulatory nexus that is heavily implicated in both ageing and senescence. Excitingly, a growing body of research has highlighted rapamycin and other mTOR inhibitors as promising treatments for a broad spectrum of age-related pathologies, including neurodegeneration, cancer, immunosenescence, osteoporosis, rheumatoid arthritis, age-related blindness, diabetic nephropathy, muscular dystrophy, and cardiovascular disease. In this review, we assess the use of mTOR inhibitors to treat age-related pathologies, discuss possible molecular mechanisms of action where evidence is available, and consider strategies to minimize undesirable side effects. We also emphasize the urgent need for reliable, non-invasive biomarkers of senescence and biological ageing to better monitor the efficacy of any healthy ageing therapy.

## 1. Introduction

The greatest risk factor for all major life-threatening diseases, including cancer, neurodegeneration, and cardiovascular disease is age. Current therapies that target each of these age-related diseases (ARD) individually have had limited success, and a cure for one specific ARD may not greatly extend healthy lifespan, as elderly patients would still be vulnerable to other ARDs. However, mounting evidence suggests that it may be possible to develop broad-spectrum treatments for the diseases of old age by targeting the underlying biological mechanisms driving ageing and its associated pathologies. Indeed, several consistent hallmarks of ageing have been identified, including telomere attrition, epigenetic dysregulation, altered proteostasis, decreased autophagy, mitochondrial dysfunction, and increased DNA damage [1]. All of these processes contribute to the onset of cell senescence, a core driver of ageing, as demonstrated by improved health and extended lifespan of middle-aged mice upon the removal of senescent cells [2]. Furthermore, it is also possible that other hallmarks of ageing, including stem cell depletion and remodelling of the extracellular matrix [1], are in fact consequences of cell senescence.

### 1.1. Senescence

Cellular senescence is a programme of essentially permanent proliferative arrest, induced by stresses including replicative exhaustion, DNA damage, oncogene signalling, ER stress, and imbalances in ribosome biogenesis [3]. At least in vitro, senescent cells show greatly enlarged cell size, altered morphology, accumulation of lipid droplets and lipofuscin-type pigments [4], and prominent actin stress fibres. Mitochondrial load increases in senescence, possibly to compensate for chronically damaged mitochondria, and lysosomal stress is evident with dyes such as senescence-associated β-galactosidase (SA-β-gal) [5]. Senescent cells exhibit chronically elevated levels of DNA damage response proteins including 53BP1 and γH2AX indicating poor DNA repair capacity, while there is also marked restructuring of the epigenome, such that CpG methylation patterns can be used as an epigenetic clock to determine biological age [6]. At the biochemical level, activation of tumour suppressor proteins p53 and/or p16^CDKN2^, together with cyclin-dependent kinase inhibitor p21^CDKN1^, leads to cell cycle arrest and the cessation of proliferation that is characteristic of senescent cells, together with resistance to apoptosis.

While the original evolutionary role of senescence may lie in development [7], wound healing [8], or as a barrier to viral infection [9], it also provides a failsafe mechanism against proliferation of tumorigenic or aged cells [10]. However, this can be detrimental to tissue integrity, as such cells can no longer contribute to wound healing or the cell turnover necessary for tissue maintenance. Moreover, senescent cells do not simply exist as passive but ineffective components of a tissue: instead, they actively alter their microenvironment through a secretory programme termed the SASP (senescence-associated secretory phenotype) [11]. This pro-inflammatory programme comprising cytokines, chemokines, growth factors, and matrix-remodelling enzymes alerts immune cells to the presence of senescent cells, which in younger organisms is thought to promote immune clearance [12]. However, with increasing age comes both an increasingly unbalanced and dysfunctional immune system, and an increased rate of senescence onset via chronic exposure to extrinsic and intrinsic damaging agents, gradual loss of homeostasis, and progressive telomere erosion. Together, these cause the accumulation of senescent cells, observed in various tissues with chronological age [5,13,14]. Pleiotropic SASP signalling also induces paracrine senescence in neighbouring cells, amplifying the senescent cell burden, and possibly driving the chronic and sterile inflammation observed in old age—a contributing factor to the development of many ARDs. Components of the SASP also participate in paracrine pro-tumorigenic signalling (e.g., IL-6, IL-8, MMP-3), promoting tumour formation and progression [11]. Several notable experiments have provided evidence for the causative role of cellular senescence in organismal ageing and age-related pathology; most convincingly, the clearance of p16-expressing senescent cells in vivo rejuvenates naturally aged mice, improving health, and extending lifespan [2].

### 1.2. mTOR Signalling in Senescence and Ageing

The serine/threonine kinase mTOR is a major regulatory nexus that integrates signals, including levels of glucose, amino acids, oxygen, growth factors, and hormones to direct cell growth and proliferation under suitable conditions. mTOR is the functional enzyme within two distinct complexes—mTORC1 and mTORC2—where it associates with several other proteins that are either distinct to each complex (e.g., Raptor/Rictor) or present in both (e.g., Deptor, mLST8 (mammalian lethal with SEC13 protein 8), see Table 1). A novel mTOR complex containing GIT1 (GPCR kinase-interacting protein 1), but lacking Raptor and Rictor, has been identified by proteomic analysis of neural stem cells and astrocytes [15], highlighting the possible variation in mTOR complex composition between somatic tissues.

mTORC1 regulates pathways central to cell growth, proliferation, survival, motility, autophagy, and protein synthesis, whilst mTORC2 has a role in regulating actin organization as well as metabolic control [16]. mTORC1 is activated by recruitment to the lysosome through the action of Rag GTPases and regulators, such as the late endosomal/lysosomal adaptor and MAPK and mTOR activator (LAMTOR/Ragulator), whereas mTORC2 is ribosomally-associated on activation by insulin-signalling, mediated through IGFR (insulin-like growth factor receptor) and IRS1/2 (insulin receptor substrate) [16], though localisation at mitochondria, the plasma membrane, ER, and lysosomes has also been reported [17] (Table 2). There is significant cross-talk between the two complexes through various positive and negative feedback loops (particularly through the kinase Akt/PKB (protein kinase B)) [16], and possibly also through competition for FKBP (FK506 binding protein) subunits [18]. Recent research using unbiased phosphoproteomics has expanded the list of known direct mTOR substrates [19,20,21] and the mTOR signalling network has been reviewed extensively elsewhere [16,22,23]. Examples of key regulators, phosphorylation targets, and biochemical and biological outcomes for each complex are summarized in Table 2.

The involvement of mTORC signalling in ageing is supported by a large body of experimental evidence. Mutations in TOR have been shown to increase the lifespan of yeast [24], *C. elegans* [25,26,27], and Drosophila [28]. Furthermore, deletion of S6K1 (ribosomal S6 protein kinase 1), which is a downstream target of mTOR, increases lifespan in female mice. Further, reduced mTOR signalling increases lifespan and reduces age-related pathologies, including motor dysfunction and loss of insulin sensitivity [29]. Notably, such findings contrast with other reports that chronic mTORC inhibition induces diabetes [30]. This finding has been attributed to differential effects on mTORC1 versus mTORC2, though in some instances loss of mTORC2 signalling also increases lifespan and improves health. For instance, in the nematode worm, reduction in mTORC2 signalling by RNAi depletion of Rictor can increase the lifespan under conditions of stress (high temperature) or high-quality food, whereas the opposite is seen at lower temperatures and on a less rich food source [31].

mTOR signalling is highly significant in senescence as well as in ageing. Notably, the proliferative arrest that characterizes cellular senescence is not accompanied by a down-regulation of growth signalling. In fact, mTOR signalling is constitutively active in senescence, resulting from replicative exhaustion, oncogene activation, and other stresses [32], and it may drive the process of geroconversion [33] i.e., the shift from proliferation to senescence without inhibition of growth. Inhibition of mTOR in cells approaching senescence reverses many of the characteristic senescence phenotypes [34] supporting a role for mTOR in driving senescence. Rather than being dramatically increased, however, mTOR signalling may instead be dysregulated in senescence; mTORC1 activity persists despite the removal of serum and amino acids in senescent but not proliferating fibroblasts, indicating constitutive activation that may be attributable to depolarization of the senescent cell plasma membrane [32].

**Figure 1:**
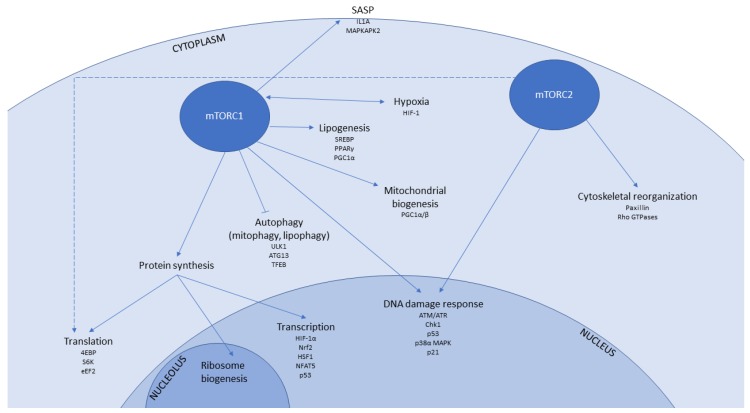
Summary of pathways targeted by mTOR signalling which are implicated in modulation of senescence and ageing. Arrows indicate that mTORC activity positively regulates the process, while bars indicate inhibition.

Both the molecular mechanisms behind healthspan and lifespan extension afforded by mTOR inhibition, and the roles of mTOR signalling in senescence are likely to be multi-factorial, as mTOR regulates a multitude of downstream signalling events (Table 2 and Figure 1). Below, we consider major biochemical pathways that are important in ageing and cell senescence that are regulated by mTORC signalling, and that may therefore be amenable to modulation by mTORC inhibitors.

### 1.3. mTOR-Associated Pathways That Contribute to Senescence and Ageing

#### 1.3.1. Transcription

mTOR signalling from both complexes can influence gene expression through interaction with a variety of transcription factors, including many involved in stress responses. For example, mTORC1 can modulate both the translational and the transcriptional activity of the hypoxia response factor HIF-1α during normoxia and hypoxia, respectively [35,36]. Furthermore, mTORC1 regulates the ROS-responsive transcription factor Nrf2 [37], as well as the heat-shock transcription factor HSF1 [38] and the osmotic stress transcription factor NFAT5 [39]. The effects of mTOR in modulating p53-dependent transcription are described in Section 1.3.7 (DNA damage response), below.

#### 1.3.2. Protein Translation

Protein translation occurs within the ribosome, a large molecular factory that is composed of functional RNAs and proteins. Ribosomal biogenesis (and hence subsequent protein synthesis) requires the coordination of transcription of ribosomal RNAs (rRNA) within the nucleolus by RNA polymerase I, protein-encoding messenger RNAs (mRNA) by RNA polymerase II and transfer RNAs (tRNA) and a further 5S ribosomal RNA by RNA polymerase III, and is positively regulated by mTORC1 signalling at multiple stages [40]. Assembly of the ribosome from ribosomal RNAs and proteins also occurs within the nucleolus. Interestingly, nucleoli are enlarged in premature ageing [41], while small nucleoli are associated with longevity [42], suggesting that enhanced ribosomal production may be associated with ageing, either as a response to imbalances in ribosomal components or as a driver through increased protein synthesis.

Protein synthesis requires not only functional ribosomes but also coordinated activity of a number of translation initiation and elongation factors. Two well-established phosphorylation targets of mTORC1 signalling are 4EBP1 and S6K, which act as regulators of translation initiation. Unphosphorylated 4EBP1 binds to and inhibits eIF4E, which is a DEAD-box helicase necessary for unwinding secondary structures at the 5′ ends of transcripts, and that serves as a critical factor in recruiting 40S ribosomal subunits to mRNAs for cap-dependent translation initiation (thought to be the rate-limiting step in protein synthesis); this inhibition is relieved by mTORC1-mediated phosphorylation of 4EBP1 [43]. S6K is activated by phosphorylation by mTORC1 [16], and S6K then phosphorylates the S6 protein, a structural component of the 40S ribosomal subunit. S6K is also involved in ribosome biogenesis and in regulating the translation of 5′TOP (terminal oligopyrimidine tract) mRNAs; rapamycin and similar rapalogues attenuate translation of mRNAs with complex 5′ UTRs especially those encoding HIF1α and VEGF [44]. The impact of mTOR signalling on 4EBP1 and S6K does vary according to cell type [45], presumably allowing for the tailoring of translational responses to a cell’s needs. Furthermore, mTOR also regulates translation elongation through activation of eEF2, which promotes the translocation of the ribosome along the mRNA. While regulation of protein synthesis has largely been attributed to mTORC1, recent evidence suggests a role for mTORC2 in co-translational processing of nascent polypeptides [46,47]. Direct activation of mTORC2 by association with the ribosome also suggests a strong link between translation and mTORC2, possibly ensuring that mTORC2 is only active in growing cells [46].

Mutations in 4EBP1, S6K, and several other components of the translational machinery can confer increased longevity, and mild restriction of protein synthesis by low dose cycloheximide can prevent induction of senescence [48]. It is possible that attenuating protein translation may prevent the production of damaged proteins by enhancing quality control to prevent translational errors, co-translational misfolding, or ER-stress, and that mTORC inhibitors, by reducing rates of protein synthesis, may prevent the formation of potentially toxic aggregates in the cell. mTOR is regulated by chaperone availability to link translation with quality control [49], suggesting that constitutively active mTOR signalling with elevated levels of translation may be detrimental to cell health. Notably, the dysregulation of protein synthesis and accumulation of protein aggregates are implicated in many age-related diseases, including diabetes and neurodegenerative Alzheimer’s, Parkinson’s and Huntington’s diseases; such dysregulation is likely to occur through a combination of high levels of translation, poor post-translational quality control, and a failure of protein breakdown through autophagy.

#### 1.3.3. Autophagy

Autophagy is a selective homoeostatic degradation pathway for cellular components, which are directed via double-membrane vesicles (autophagosomes) to lysosomes for degradation. Autophagy is activated in response to nutrient limitation and is suppressed by mTOR activity, through the inhibitory phosphorylation of the autophagy-initiating kinase ULK1 (ATG1) [50], ATG13, and lysosomally-located TFEB (reviewed in [51]).

The published literature contains some discrepancies about the association between autophagy and ageing. In acutely triggered oncogene-induced senescence, autophagy activation has been observed [52], possibly to rebalance the proteome for transition into a senescent state. However, in almost every other model described, decreased autophagy is linked to ageing. For instance, several proteins that are required for autophagy (Atg5, Atg7 and Beclin 1) are downregulated in normal human brain ageing [53] and in osteoarthritis (ULK1, Beclin 1 and LC3) [54], while knock-in of an activated form of Beclin 1 delays the onset of cardiac and renal fibrosis in normally ageing C57/BL6 mice, and even rescues the short lifespan of Klotho mutant mice [55]. Reduced autophagy has also been observed alongside mTOR activation in senescence resulting from treatment with the genotoxin adriamycin, and co-treatment with the autophagy inhibitor Bafilomycin A1 further increased the proportion of cells that are positively stained for SA-β-gal, a marker of senescence [56]. Increased autophagy has been suggested to mediate the pro-longevity effects of caloric restriction (CR), as inhibition of autophagy prevents CR-mediated anti-ageing effects [57]. Activation of autophagy by spermidine decreases immunosenescence and improves the response to influenza vaccination in mice [58]. Decreased autophagy in ageing may limit the removal of dysfunctional organelles, such as mitochondria, and lead to the accumulation of protein aggregates in neurodegenerative disorders. Autophagy has also been implicated as a mechanism for the antagonistic effects of SIRT6 expression on senescence in rat nucleus pulposus (NP) cells in a model of invertebral disc degeneration (IDD); SIRT6 expression declines in senescent NP cells, but when overexpressed, it attenuates senescence, with this effect being dependent on activation of autophagy and mTOR inhibition [59]. Furthermore, an acetylcholine esterase inhibitor designed as a potential Alzheimer’s treatment was shown to induce senescence in MCF-7 breast cancer cells, while simultaneously inducing the onset of autophagy but blocking autophagic flux, leading to the production of single-membrane autolysosomes with non-degraded cargo [60]. Hence, initiation of autophagy with failure of autophagosome fusion with lysosomes for complete protein and organelle recycling may contribute to cell stress and senescence. These results taken in combination underline the complex role of mTOR signalling in regulating autophagy in senescence, and additionally highlight the inadequacy of usual markers of autophagy (autophagosome number or LC3-II/LC3-I ratio) as readouts for activation of such a complex pathway that is subject to further downstream regulation. On balance, we suggest that reactivation of autophagy through mTORC1 inhibition is likely to be beneficial in many different diseases that are associated with ageing, as discussed in Section 2 below.

#### 1.3.4. Mitochondrial Function and Biogenesis

The progressive decline of mitochondrial efficiency in senescence represents a key hallmark of ageing [1]. Senescent cells accumulate dysfunctional mitochondria, with both reduced oxidative phosphorylation efficiency and increased ROS production [61,62]. Mitochondrial dysfunction is itself a driver of cell senescence, with senescent cells exhibiting an increased mitochondrial load and increased oxygen consumption [63]. The relationship between mitochondrial dysfunction and senescence may be inter-dependent, as the chronic DNA damage response of senescent cells also promotes mitochondrial dysfunction [64]. Furthermore, mitochondrial fission and fusion events are altered in senescence, resulting in increased connectivity of the mitochondrial network [65]. As well as the oxidative stress that is caused by dysfunctional mitochondria, mitochondrial nitrosative stress (excess *S*-nitrosylation) is implicated in senescence, through enhanced *S*-nitrosylation of proteins regulating mitophagy and mitochondrial dynamics [66].

mTOR provides a critical link between the energy balance of the cell and mitochondrial load, regulating both mitochondrial biogenesis and mitophagy. Biogenesis is controlled through several mechanisms, including PGC-1-β-dependent mitochondrial biogenesis and preferential translation of nuclear-encoded mitochondrial-related mRNAs via the relief of 4EBP inhibition [67], with mitochondrial oxidative function controlled through the YY1-PGC-1α transcriptional complex [68].

#### 1.3.5. Hypoxia

The transcription factor HIF-1, active under hypoxic conditions, has been linked to ageing in *C. elegans*, with increased and reduced activity both causing lifespan extension, dependent on context. mTORC1 signalling is inhibited on HIF-1 activation, through transcription of REDD1, which activates the TSC1/TSC2 complex, resulting in mTORC1 inhibition. Conversely, high oxygen tensions lead to mTORC1 activation, while reactive oxygen species (ROS) may specifically activate mTORC2 [69,70] to promote survival under oxidative stress. However, high Rheb activity in many cancers leads to hyperactive mTOR signalling and increased HIF1 activity, resulting in the upregulation of VEGF and high vascularisation of the tumour [71]. Hence inhibition of mTORC through rapalogues or second-generation mTOR inhibitor ATP mimetics may have a beneficial impact on cancer through blocking this pathway. Whether this has direct relevance to ageing remains to be determined, though it has been suggested that ageing induces an mTOR-dependent pseudo-hypoxic state with high HIF1 and lactate production under normoxic conditions [72,73], which may be amenable to modulation by mTORC inhibition.

#### 1.3.6. Immunomodulatory Signalling

A common feature of age-related pathologies is chronic sterile inflammation. The secretory phenotype (SASP) of senescent cells, through which pro-inflammatory mediators are released to stimulate clearance by immune cells, may be the source of such inflammation. The SASP has pleiotropic signalling effects, exhibiting not only paracrine immunomodulatory signalling, but also autocrine and paracrine pro-senescence, and paracrine pro-tumorigenic signalling. Therefore, the SASP may amplify the senescent cell burden of an elderly individual, exacerbate tissue dysfunction, and stimulate age-related tumorigenesis. The SASP is at least partially regulated by mTOR, possibly through feedback loops of IL1A translation or MAPKAPK2 signalling, and it can be suppressed while using rapamycin or Torin [74,75], or MAP kinase inhibitors [76]. These findings conflict with earlier studies showing the central importance of mTOR in innate immunity, specifically in the production of anti-inflammatory IL-10 and the suppression of pro-inflammatory cytokines IL-21 and IL1β. Rapamycin and Torin are also reported to suppress the anti-inflammatory effects of circulating glucocorticoids [77]. Furthermore, transplant patients receiving mTORC inhibitors showed more than double the expected rate of non-infectious fever [78], suggesting excess inflammation. It is possible that these important and marked discrepancies relate to dosage, with pro-inflammatory effects of mTORC inhibition being caused by high dosage, while anti-inflammatory suppression of the SASP may be achievable at much lower doses.

#### 1.3.7. DNA Damage Response

Following DNA damage, cell cycle progression is halted through the activation of multiple checkpoints and cyclin-dependent kinase inhibitors. The damage-responsive ATM/ATR kinases phosphorylate and activate mTORC, which can then phosphorylate Chk1, leading to proliferative arrest at either S phase or G2/M; mTORC2 is specifically implicated in this arrest, at least in breast cancer cells [79]. In addition to Chk1, components of the mTOR/S6K axis are also phosphorylated by p38α MAPK following DNA damage. While mTOR activity can itself be modulated by the tumour suppressor protein p53 (e.g., through p53 transcriptional targets such as TSC2, AMPK, and REDD1 [80]), p53 activity is sensitive to mTOR signalling; mTORC1 can enhance the translation rate of p53 [81,82] or activate p53 through S6K1-dependent phosphorylation of and binding to MDM2, which releases p53 from inhibition [83] so that it can act as a transcription factor for repair factors, such as Gadd45 or pro-apoptotic factors Bax and PUMA (reviewed in [84,85]). Moreover, mTOR activity enhances p53-dependent transcription of p21^CDKN1^ and induction of senescence [86], a possible molecular explanation for the importance of mTOR in geroconversion.

The importance of mTORC in DNA damage responses suggests that mTORC inhibitors may be beneficial in cancer by sensitizing cells to genotoxic agents, though conflicting results have also been reported [21]. Very recent work suggests that the DNA damage response is defective in cells with hyper-activated mTORC1 signalling that lack the LKB1 tumour suppressor [87]. Chronic persistent DNA damage—and constitutively active mTOR—are also features of senescent cells. Hence, mTOR inhibitors may alleviate the burden of DNA damage on ageing, though their impact on cell cycle control should be closely monitored.

#### 1.3.8. Lipid Metabolism

As a central regulator of cellular growth, mTOR also regulates lipid metabolism, through affecting lipogenesis as well as lipolysis and lipophagy. mTORC1 signalling activates SREBP transcription factors that drive fatty acid (FA) biosynthesis for lipogenesis [88] through an indirect mechanism, whereby mTORC1-phosphorylated Lipin-1 is no longer translocated to the nucleus [89,90]. (Lipin-1 is itself a phosphatidic acid phosphatase that is involved in triacylglycerol synthesis). Furthermore, PPARγ is a SREBP transcriptional target and mTORC1 may also regulate PPARγ activity [91], as well as inhibiting PPARα and PGC1α, which further regulate fatty acid oxidation [92]. PPARα activity is reduced in aged mice (alongside increased mTORC1 activity), but the inhibition of mTORC1 is sufficient to prevent the loss of PPARα activity [93]. The autophagic recycling of lipid droplets for degradation (lipophagy) is suppressed by mTORC1 signalling. Furthermore, decreased lipolysis and triacylglycerol accumulation are observed following the knockdown of 4EBP1 and 4EBP2, suggesting a role for mTORC1 signalling in lipolysis [94].

Senescent cells exhibit dysregulated lipid metabolism, characterized by increased uptake and accumulation of lipids, with coincident increase in oxidative damage to lipids. Notably, the addition of specific lipids such as triglycerides and cholesterol to delipidized media can induce senescence in vitro. This finding suggests altered lipid metabolism as a possible driver of senescence [95], potentially through adding to the ROS burden via β-oxidation of fats, and through lipid peroxidation producing aldehyde end-products, which can cause DNA and protein adducts [95]. Treatment with mTOR inhibitors in vitro has been shown to reduce lipid droplet accumulation in senescent cells [33].

### 1.4. Rapamycin and Other mTOR Inhibitors

Rapamycin is the natural macrolide antibiotic lactone that is produced by *Streptomyces hygroscopius,* discovered in soil samples from Easter Island, and initially noted for inhibiting the proliferation of yeast [96]. At high doses (e.g., 5 mg/day), rapamycin has immunosuppressive effects and it is FDA-approved for prevention of transplant rejection [97]. It is also in clinical use or in trials for a large number of cancers where mTORC signalling appears to be a key factor in promoting and/or sustaining oncogenic transformation (see Section 2.8 below). Reported side-effects of chronic administration include ulceration of mucosal tissues, haematological abnormalities, induction of insulin insensitivity, obesity, and diabetes, though these adverse effects may be largely dose-dependent.

As discovered through *S. cerevisiae* genetic screens [98], rapamycin mechanistically acts by binding the protein FKBP12, producing a complex that can bind the FRB region of mTOR and partially occlude the active site of mTOR kinase in the mTORC1 complex [99]. This induces cellular effects, including a decrease in protein synthesis, increase in autophagy, and inhibition of cellular growth [100]. Rapamycin does not inhibit the phosphorylation of all mTORC1 substrates equally—it completely inhibits S6K1 phosphorylation, while only partially blocking 4EBP1 phosphorylation [45]. A crystal structure of mTOR, rapamycin, and FKBP12 [101] suggests that this may be due to differential substrate access to the kinase active site, controlled by the mTOR FRB domain, though differential substrate quality (i.e., degree of divergence from the consensus sequence of the phosphorylation site) could also be important.

Structural and functional analogues of rapamycin (known as rapalogues) that also act by allosterically modulating the enzyme have been developed to improve bioavailability and pharmacokinetics, including drugs such as everolimus (RAD001). These agents also act by recruiting the immunophilin/prolyly isomerase FKBP12 to mTORC1.

By contrast to mTORC1, mTORC2 is not particularly sensitive to inhibition by rapamycin or rapalogues, though chronic administration does impact mTORC2 signalling [102], either through feedback via the insulin signalling pathway, and/or through competition for key subunits FKBP12, 51 and 52, which may set different thresholds for rapamycin sensitivity between the two complexes [18]. In human cells in culture, the ‘chronic’ effect on mTORC2 is observed as little as 24 h after drug treatment, though metabolic effects in animals and human patients require more prolonged treatment (over weeks or months). mTORC2 inhibition is implicated in impaired glucose homeostasis, insulin insensitivity, and diabetes, though studies on worms with tissue-specific RNAi have suggested that it is loss of mTORC2 activity, specifically in the intestine that results in the dysregulation of glucose metabolism [31]. It is important to note that such studies often rely on phosphorylation of mTORC2 target Akt on S473 as a readout of mTORC2 activity, but this site on Akt may also be targeted by kinases IKKε, TBK1 [103], and DNA-PK [104], potentially skewing the interpretation of mTORC2-specific effects.

Second-generation mTOR inhibitors have been developed, primarily as anti-cancer agents to target the hyperactive mTOR observed in many cancers [105]. These drugs compete with ATP for the active site of the mTOR kinase, and hence are effective in inhibiting both mTORC1 and mTORC2. Some agents have extremely high specificity and selectivity for the mTORC kinase. For example, AZD8055 has 1000-fold greater inhibitory effect on mTORC than on other PI3 kinases [106], whereas others (e.g., BEZ235) have dual inhibitory effects on both mTORC and PI3K [107], with a 3–5 fold higher K_d_ for damage response kinase ATR [108]. While these ATP-competitive inhibitors exhibit more potent apoptotic effects in vitro compared with rapalogues, and a number of such agents have been tested in clinical trials for safety, larger scale trials have not yet demonstrated greater efficacy than current best treatment regimens [105]. Therefore, drugs such as AZD8055, AZD2014, and WYE354 have not yet received FDA approval. The differential specificities of rapalogues and second generation mTORC inhibitors have proven useful in primary research to dissect the effect in senescence of mTORC1 inhibition (rapalogues) versus dual mTORC1/2 inhibition (competitive ATP mimetics) [34]. The major classes of mTOR inhibitors and other pathway modulators are listed in Table 3.

## 2. Ageing and Age-Related Pathologies Amenable to Treatment by mTOR Inhibition

### 2.1. Ageing

A landmark study from 2009 in which rapamycin was fed to middle aged mice provided the first evidence that any small molecule drug, taken orally, could significantly extend both the mean and maximum lifespan in mammals [126]. In this multi-centre, large cohort study of genetically heterogeneous (UM-HET3) mice, rapamycin delayed the ageing of 20-month old male and female mice. Further studies have not only validated these results, but have demonstrated that rapamycin improves health, in terms of lower incidence or decreased severity of age-related disease, as well as prolonging life [127]. Below, we assess the impact of mTOR inhibition on a number of age-associated diseases and pathologies, collating findings from model systems and human clinical trials.

### 2.2. Immunosenescence

The immune system undergoes a functional decline with age that both contributes to organismal ageing through decreased senescent cell clearance, and also compromises its ability to fight infection. The term immunosenescence is specifically associated with a decline in the haematopoietic stem cell proliferation compartment, a higher proportion of exhausted, PD-1^+^ lymphocytes, an inverted CD4/CD8 ratio (<1), a low number of B cells, and seropositivity for cytomegalovirus (CMV) [128]. Age is associated with a high mortality rate from infectious disease, thought to be a direct consequence of loss of immune function. Activation of autophagy has been shown to rejuvenate the immune system in mice [58]; since mTOR activity inhibits autophagy, it follows that mild inhibition of mTOR could be beneficial for immune function with increasing age. Deriving an appropriate dose is critical, as at high doses rapamycin is immunosuppressive, blocking both the protein synthesis and cell division that are required to mount an adaptive immune response.

In mouse models, increased immune activity against both viral and bacterial pathogens has been observed on mild mTOR inhibition [129], suggesting that it is possible to improve at least some aspects of the ageing immune system with low dose mTOR inhibitors. Furthermore, a placebo-controlled, randomized, double-blind human clinical trial of over 200 elderly volunteers has shown similar results [110]. Volunteers were assigned to one of three regimes of the mTORC1 inhibitor RAD001 (everolimus—low: 0.5 mg daily or 5 mg weekly; high: 20 mg weekly) for a six-week period, followed by a two-week drug-free interval. These volunteers were then challenged with the seasonal influenza vaccine. Though the relatively small size of the study impeded powerful statistical analysis, the two low-dose RAD001 regimens improved immune function without causing serious side effects. Patients produced a broader and more powerful immune response, with improved HSC function and a decreased proportion of PD-1^+^ lymphocytes. The increased breadth of the immune response was particularly promising; older individuals are more likely to die from influenza than younger people, but they generally produce a narrow, weak response to vaccination. Despite the lack of a young control population in the study, the improved response is thought to correspond to a rejuvenated immune system. In a subsequent follow-up study using combined BEZ235 and RAD001 treatment, again for just six weeks, better infection control was reported in older adults for a year after treatment ended [111]. Given the important role of the immune system in cancer surveillance and senescent cell clearance, it would be very interesting to test whether such a rejuvenated immune system is better equipped to clear senescent or tumorigenic cells in vivo.

### 2.3. Age-Related Neurodegeneration

mTOR hyperactivation is associated with cognitive deficit and brain dysfunction, as seen in Tuberous Sclerosis (TS), where the loss of TSC1/2 prevents negative regulation of mTOR. Hence, mTOR inhibition is being trialled for TS treatment, with beneficial results being reported (reviewed in [130]). Lifelong rapamycin administration to mice prevents the usual age-related decline in cognitive function, thought to be through suppression of IL1β [131]. Neurodegenerative diseases that are characterized by accumulation of abnormal protein aggregates (Alzheimer’s disease, Parkinson’s disease, and Huntington’s disease) are further candidates for treatment with mTOR inhibitors. Not only does mTORC1 exert tight control over protein synthesis and degradation (autophagy) through 4EBP1/S6K, ULK1, and SCF/FBW8, but the mTOR pathway is involved in regulating the inflammatory responses that are known to be involved in the progression of neurodegeneration; it may also contribute to an energetic deficiency observed in such diseases. Conversely, however, the mTOR pathway has been proposed to regulate synaptic plasticity and memory consolidation, through the control of actin reorganization by mTORC2 [132], and neuronal Rictor knock-out mice do indeed show cognitive effects due to alterations in actin reorganisation needed for dendritic spine growth and formation of memories [133]. However, human trial data suggest that pharmacological inhibition, which is not equivalent to total loss of mTORC2, is if anything supportive of brain function since patients taking everolimus for immunosuppression after heart transplantation actually showed improvements in memory and concentration in comparison to those on calcineurin inhibitors [134].

#### 2.3.1. Alzheimer’s Disease

Alzheimer’s disease (AD) is the most prevalent neurodegenerative disease, which is characterized by accumulation of aggregated extracellular amyloid β (Aβ) plaques and intracellular neurofibrillary tangles composed of tau protein. Neuronal loss and brain atrophy worsen with disease progression. mTOR signalling has been implicated in AD pathogenesis: evidence from human post-mortem exams suggests that mTOR activity is upregulated in AD brains compared to age-matched controls, as levels of phosphorylated mTOR, p70S6K and eIF4E are all increased in AD [135]. This upregulation of mTOR signalling could be mediated via Aβ accumulation, which may activate the PI3K/AKT pathway, and in turn, increased mTOR signalling has been linked to the development of tau pathology [136]. Aβ upregulates mTOR and mTOR is thought to increase levels of Aβ (reviewed in [137]), potentially generating a positive feedback loop in disease progression.

Rapamycin has been shown to prevent cognitive decline in the AD-Tg mouse model of Alzheimer’s disease [138,139,140], and even to reverse already established memory deficits [141], though these effects were limited to mild cognitive decline before widespread plaques and tangles were observable. Improvements in memory and cognition with rapamycin or tersolimus treatment correlated with improvements in the three major hallmarks of AD (Aβ plaques, tau tangles, and microglia activation) [139,140,141]. A genetic mouse model lacking one mTOR gene copy in the brain exhibited reduced Aβ deposits and rescued memory deficits [142], hence reduced mTOR activity associates with cognitive improvement. It is likely that treatment must happen prior to major amyloid or tau deposition, as cognitive improvements are seen in mice on whole-life but not late-life administration of rapamycin—i.e., a therapeutic window exists, though it is not yet known what constitutes the point of no return.

Though the mechanism of improvement is still unclear, it is possible that decreased protein synthesis may avoid the build-up of toxic Aβ, or that the induction of autophagy through mTORC1 inhibition may result in the removal of protein aggregates. Healthy neurons have highly efficient and active autophagy, but this decreases with age (reviewed in [143]). In the mouse models where rapamycin was shown to decrease levels of Aβ, autophagy induction was necessary [138]. Further, in rapamycin-treated AD-Tg mice brains, increased localization of Aβ into lysosomes was detected, suggesting a more active degradation of these peptides [138], and the decrease in Aβ levels induced by rapamycin could be prevented by blocking autophagy. Hence, mTOR inhibition leading to increased autophagy may be beneficial in treating neuropathies that are associated with protein aggregation. Other components of the mTOR signalling cascade are also implicated in neurodegeneration, including GSK3, overactivity of which results in decreased lysosomal acidification. Hence, GSK3 inhibitors (such as peptide L803-mts) present a novel alternative to mTORC inhibition in AD, and appear to be active in the 5xFAD mouse model of AD [144].

#### 2.3.2. Huntington’s Disease

Huntington’s disease (HD) is a neurodegenerative disorder where a genetic mutation causes an expansion of the polyglutamine tract within the Huntingtin protein (HTT), resulting in protein aggregation. As mTORC1 signalling suppresses autophagy, which is responsible for recycling protein aggregates, it has been implicated in HD pathology. Counter-intuitively, however, mTORC1 activation may actually be beneficial: in HD mouse models with increased mTOR activity, motor performance was improved relative to controls, coincident with improved mitochondrial function, cholesterol synthesis, and decreased HTT abundance. Further, phosphorylation of S6 was actually decreased in human HD patients as compared to controls, further suggesting a complicated association between mTOR signalling and HD [145].

#### 2.3.3. Parkinson’s Disease

Parkinson’s disease (PD) is a progressive age-associated neurodegenerative disorder associated with the death of neurons in the substatia nigra. It manifests as loss of motor coordination, often associated with mood disturbance and in many cases followed by dementia. Current treatment is symptomatic, using L-DOPA to reinforce failing dopaminergic signalling. Though a number of genes are associated with PD, there is little overall understanding of the etiology, but lysosomal dysfunction (allowing for a build-up of intracellular α-synuclein as Lewy bodies) is implicated. Failure of mitophagy, through defects in PINK1/Parkin, may also be important, and defective mitochondria are observed in PD [146].

mTORC1 has been suggested to be neuroprotective in PD, and consistent with this, suppression of mTORC1 signalling by several routes (AMPK, PTEN, or REDD1 activation, or rotenone treatment) results in neuronal cell death in models of PD [147,148]. Moreover, L-DOPA, the current symptomatic treatment of PD, activates mTORC1, supporting the idea that mTORC1 activity is beneficial. However, the opposite has also been reported: elevated mTORC signalling (by deletion of the gene Engrailed, or exposure to paraquat) leads to neuronal apoptosis, suggesting that a balance of mTORC activity is required for neuronal health.

To achieve this balance, mTORC inhibition is being explored as a possible treatment route for PD. Rapamycin has been shown to overcome dyskinesia in mice, which is a major side effect of treatment with L-DOPA, without interfering with the therapeutic effects of L-DOPA [149], while a number of other studies have also demonstrated benefits of rapamycin use in PD (reviewed in [150]). As in AD, other mTOR pathway factors, such as GSK3, might present therapeutic targets, particularly as lysosomal function appears important. It will be interesting to determine if mTORC inhibition promotes autophagic clearance of aggregated α-synuclein and/or dysfunctional mitochondria, and whether this is enhanced by co-treatment with GSK3-inhibiting peptides. However, it has been argued that specific pro-autophagic interventions may provide an even better therapeutic outcomes than global autophagy stimulation [151].

### 2.4. Age-Related Blindness: AMD

Age-related macular degeneration (AMD) is the most common cause of blindness in the Western world, whereby retinal damage leads to loss of vision in the centre of the visual field (macula). In senescence-accelerated OXYS rats, rapamycin administration in food decreased the incidence and severity of AMD-like retinopathy and prevented the destruction of ganglionar neurons in the retina [152]. These promising results accelerated rapamycin as an AMD therapeutic through to clinical trials, however conflicting results have since been produced, potentially because of dosing issues. For example, one small phase II clinical trial administered 440 μg rapamycin to one eye every three months for 24 months to eleven patients with an advanced form of dry AMD, but it was terminated early after finding that treatment may be detrimental to visual acuity [153]. High dose rapamycin is known to elicit unwanted side effects, so it is unfortunate that such high dosage trials have been designed and conducted, with negative outcomes, as they are likely to reinforce clinical prejudice against use of mTOR inhibitors for non-life-threatening illness. Full dose-response trials to obtain maximal benefit with minimal side effects are still needed, particularly as AMD treatment options are limited and pharmacological therapies should provide a cheaper and more accessible option to the successful stem cell treatments recently reported [154].

### 2.5. Musculoskeletal Disorders

#### 2.5.1. Sarcopenia and Muscle Wasting

Structural and functional remodelling of skeletal muscle throughout ageing causes sarcopenia, a muscle-wasting syndrome that results in frailty. Muscle loss is consistently observed in premature ageing syndromes and associated with mTOR signalling. For example, muscle-derived stem/progenitor cells (MDSPCs) from the premature ageing Ercc1^−/Δ^ mouse show upregulated mTOR signalling and are defective in differentiation. Treatment with rapamycin improved myogenic differentiation, with increased levels of autophagy being detected in the isolated cells [155]. Hutchinson-Gilford progeria syndrome (HGPS), which is a human early onset premature ageing syndrome, is also associated with musculoskeletal abnormalities. HGPS results from a splice site mutation in the lamin A (LMNA) gene, leading to the production of an aberrant lamin protein termed progerin, though even in normal individuals, progerin accumulates during ageing, and is associated with vascular pathology. Rapamycin treatment can induce autophagy and reduce phenotypes of senescence induced by progerin in cell culture models of HGPS [156]. Based on such studies, everolimus has now been included in a clinical trial for 17 children with HGPS [157].

The muscle loss in premature ageing HGPS is highly similar to that seen in various other laminopathies, including Emery-Dreifuss muscular dystrophy, Limb-girdle muscular dystrophy, and dilated cardiomyopathy. mTORC1 is implicated in these LMNA-related dystrophies: both *Lmna^H222P/H222P^* and *Lmna*^−/−^ mice show aberrant mTORC1 signalling [158]; *Lmna*^−/−^ mice specifically showed increased mTORC1 signalling in cardiac and skeletal muscle, with impaired cardiac autophagy, while rapamycin treatment enhanced cardiac and skeletal muscle function and survival in the mutant mice [159].

Targeting mTORC1 signalling is the only therapeutic avenue yet explored for laminopathies that has promise against both dystrophic and progeroid laminopathies [160], but it has yet to be tested in sarcopenia. However, as a note of caution, patients taking rapamycin for more than six months for the treatment of renal cell carcinoma or paracrine neuroendocrine tumours demonstrated an increase in sarcopenia [161], a worrying finding as sarcopenia is predictive of outcomes in cancer patients. Longitudinal rapamycin studies in healthy subjects, such as those that are ongoing in companion dogs [162], are needed to inform on whether low dose mTOR inhibition may be able to delay or even prevent the onset of sarcopenia.

#### 2.5.2. Osteoporosis

Osteoporosis is a common ARD that is characterized by loss of bone density, causing fragility. Falls, as a consequence of co-morbid sarcopenia and age-associated changes to vision and balance perception, often result in hip fractures, and a high number of elderly fracture patients die within six months of pneumonia (exacerbated by co-morbid immunosenescence) [163,164]. Increased activity of osteoclasts, which mediate bone resorption, together with decreased osteoblast activity, is frequently seen in multiple forms of bone loss (osteoporosis, rheumatoid arthritis, and cancer-induced bone loss). mTOR signalling regulates osteoclast differentiation by altering ratios of the LIP/LAP isoforms of transcription factor C/EBPβ [165], which enhances osteoclastogenesis. In mouse models and human cells, inhibition of mTORC1 signalling lowers the activity of the translation initiation factor eIF4E, in turn diminishing expression of the LIP isoform by inhibiting translation re-initiation. This increases the LAP to LIP ratio and inhibits osteoclastogenesis, hence rapamycin treatment limits bone resorption [166,167]. Furthermore, the mTORC1 inhibitor everolimus inhibits bone loss in an experimental rat model of osteoporosis induced by ovariectomy [168].

#### 2.5.3. Rheumatoid Arthritis

Rheumatoid arthritis (RA) is a chronic autoimmune disease characterized by inflammation in joints. Highly effective treatments for RA include methotrexate and infliximab, but these have limited utility in elderly patients because of underlying renal insufficiency; factors such as transport/mobility difficulties also limit attendance at treatment centres for regular antibody infusion. Hence, a safer therapy is required in this patient cohort, which may be provided by mTOR inhibitors. Active mTOR signalling has been detected in synovial tissue from RA patients, and is crucial for joint destruction in experimental arthritis [169]. Such results appear to be relevant to human joints: in a recent proof-of-concept study (a multi-centre, randomized, double-blind study of 121 patients with RA), 6 mg everolimus daily for six months, in combination with methotrexate, showed improved clinical efficacy when compared with methotrexate alone, as well as causing few side effects [170].

Osteoarthritis (OA) is another ARD also characterized by joint inflammation but is thought to be caused by mechanical stress. Senescent cells have been detected in OA joints (Clinicaltrials.gov identifier NCT03100799), and SASP secretion of collagenase and other metalloproteases is likely to impact significantly on joint integrity. Hence, mTOR inhibition could also be beneficial in OA, by targeting constitutively active mTOR in senescent cells. Intraperitoneal administration of rapamycin reduced cartilage destruction and synovitis in experimentally-induced osteoarthritis in mice [171]; this may occur at least in part through increased ULK1-mediated autophagy and through the suppression of MMP secretion by chondrocytes (reviewed in [172]). OA presents an ideal opportunity for intervention as intra-articular drug administration should avoid potential side-effects associated with systemic mTORC inhibitor treatment.

#### 2.5.4. Diabetic Bone Fragility

Increased bone fragility is also seen in Type 1 and Type 2 diabetes mellitus (T1DM and T2DM), with increased cortical porosity and decreased cortical area in T2DM. Unlike other age-related bone pathologies, such as osteoporosis, diabetic bone fragility is not associated with decreased bone mineral density, nor does it impact on the balance between bone formation and bone resorption, but instead both bone remodelling and turnover are compromised (reviewed in [173]). This appears to arise from a combination of factors, including alterations in stem cell differentiation, glycation of collagen leading to decreased bone toughness [174], calcification of vascular smooth muscle cells though a RAGE-mediated MAPK-TGFβ-NFκB axis that increases fracture risk (at least in T1DM) through defective bone microvasculature [175], and deficits in muscle-dependent production of IL-6 on exercise that usually allow for bone to adapt to mechanical loading [176]. The decrease in bone turnover is likely to diminish capacity for microfracture repair, leading to a higher incidence of overt fractures. Notably, it has been suggested that the anti-diabetic drug metformin is protective of bone in diabetes by inhibiting adipogenesis that would otherwise be driven by mTOR/S6K signalling [177] and by lowering RAGE signalling [178]. Hence, metformin may support bone strength by acting as an mTOR pathway inhibitor, albeit indirectly.

### 2.6. Cardiovascular Disease

Cardiovascular disease is the leading cause of death in developed nations and its incidence increases with age. A number of studies have shown beneficial effects of rapamycin on cardiovascular disease in mice: for example, rapamycin has been shown to attenuate pressure overload-induced cardiac hypertrophy [179], to regress established cardiac hypertrophy and improve cardiac function [180], and to suppress experimental aortic aneurysm growth [181]. Recent studies have elaborated on this research. In female 24 month-old C57BL/6J mice fed rapamycin for three months, the greatest benefit measured was in cardiac health, with reversal or attenuation of age-related cardiac decline. Specifically, rapamycin appeared to slow or reverse the progression of age-related hypertrophy, and ventricular function of the ageing heart was also improved [182]. Through RNA-seq analysis, validated at the protein level and with bioinformatics analysis, it appeared that rapamycin reduced age-related sterile inflammation in the heart, while promoting the expression of RAD (Ras associated with diabetes), which mediates anti-hypertrophic signalling and enhances cardiomyocyte excitation-contraction coupling [183]. Caloric restriction and rapamycin treatment (both for 10 weeks) were also shown to rejuvenate the ageing mouse heart [184], with quantitative comparative proteomics revealing an age-dependent decrease in proteins that are involved in mitochondrial function, together with an increase in glycolytic enzymes, which could be reversed by either CR or rapamycin treatment. Improvements in mitochondrial function were implicated in the mechanism, as the mitochondrial proteome was rejuvenated [184], which is consistent with the known action of mTORC1 in mitochondrial biogenesis, and the contribution of mitochondrial accumulation to senescence. Hence, rapamycin could act both to suppress excessive mitochondrial biogenesis and to activate mitophagy. The authors did not observe any increase in autophagy by rapamycin or CR; instead, they observed a reduction in protein oxidative damage, alongside reduced protein turnover. Better preserved protein quality and slower turnover following CR or rapamycin treatment may therefore re-balance the oxidative phosphorylation to glycolysis shift usually seen in aged mice, though the impact of either treatment on cardiomyocyte senescence has not been analysed. It is of note that improved cardiovascular function was also the most marked outcome of the first year of a trial feeding rapamycin to companion dogs [162], thus reinforcing the potential for rapamycin to treat cardiovascular disease. It is possible that the mechanism here is through induction of autophagy by ULK1 upregulation on mTORC inhibition, as cardiac fibrosis is also decreased in older mice on the activation of autophagy by disrupting the Beclin 1-Bcl2 interaction [55]—alternatively or in addition, decreased inflammation by suppression of the SASP is also a potential mechanism.

mTOR inhibitors are also promising treatments for myocardial ischaemia/reperfusion (I/R) injury, for which diabetic patients are at especially severe risk. While the dosage and timing of administration may be critical for beneficial effects, rapamycin treatment has been shown to reduce infarct size after I/R injury in diabetic mice, through facilitating opening of mitochondrial ATP-sensitive potassium channels [185] with the effect also being dependent on STAT3 (signal transducer and activator of transcription 3) [186,187]. Improvements in oxidative stress, cytoskeleton organization, and glucose metabolism on rapamycin treatment have also been implicated in the mechanism [188].

Furthermore, rapamycin-eluting stents are now in widespread clinical use in coronary angioplasty to treat cardiovascular disease, after being approved in Europe in 2002 on the basis of very promising clinical trial results [189]. In this context, rapamycin may benefit coronary function by restricting cell proliferation and thus preventing fibrosis that could block the artery; everolimus is now also in clinical trials for this use. To date, therefore, mTOR inhibition appears to be a safe and effective intervention to improve cardiovascular function during ageing.

### 2.7. Kidney Disease

#### 2.7.1. Adult Polycystic Kidney Disease

Age-related incontinence is a common cause of depression and isolation in the elderly. A possible heritable disease model for this condition, adult polycystic kidney disease, which is also known as autosomal-dominant polycystic kidney disease (ADPKD), is the most common heritable kidney disorder, with a prevalence of between 1/400 and 1/1000. Mutations in two genes are responsible for the condition: PKD1 (85% of cases—severe, early onset) and PKD2. PKD1 codes for polycystin-1, a membrane receptor protein, while PKD2 codes for polycystin-2, a Ca^2+^-permeable channel that binds PKD1. Polycystins are involved in maintaining a differentiated epithelium in the kidney, liver and pancreas, but when mutated, excessive epithelial proliferation results in renal cysts. Mechanistically, they play a role in signalling—there are direct physical interactions between the cytoplasmic tail of polycystin-1 and tuberin, the product of the TSC2 gene, which regulates mTOR [190]. As mTOR signalling is therefore regulated by polycystin-1, and mTOR signalling is increased in murine models and in human ADPKD, mTOR activation may contribute to renal cyst expansion through excessive tubular epithelial cell proliferation. Hence, mTOR inhibition may be beneficial, and rapamycin has been shown to decrease proliferation in cystic and non-cystic tubules, to inhibit renal enlargement and to prevent the loss of kidney function in the Han:SPRD rat model of ADPKD [191,192,193]. While this model results from mutations in genes other than PKD1 and PKD2, rapamycin treatment was also effective in a more human-orthologous mouse model of conditional inactivation of PKD1 [194]. Still, both models exhibit early-onset, rapidly progressive disease, whereas human ADPKD is characterized by complex, slow, and heterogeneous progression. Therefore, retrospective analyses of human ADPKD patients after renal transplantations have been very informative. Using MRI-determined increases in kidney volume as a marker of disease progression, rapamycin-based regimens showed significantly reduced cystic kidney volumes when compared to alternative treatments [190,195,196]. Clinical trials using rapamycin to treat ADPKD have however produced varied results [197,198,199], though they may have been impeded by small sample size, reliance on poor markers of clinical progression, short follow up time for such a slow-progressing disease, and insufficient rapamycin doses [200].

#### 2.7.2. Diabetic Nephropathy

High doses of rapamycin used for immunosuppression in renal transplantation and cancer are associated with type II diabetes [30]. However, there is some evidence that low doses of rapamycin may have therapeutic benefit in the treatment of diabetic nephropathy (DN), which is one of the major complications of both type I and II diabetes [201] that currently has very limited treatment options.

In diabetes, hyperglycaemia increases mTOR activity through activation of Akt and inhibition of AMPK, which has consequences for the development of podocytes, critical in production of the renal filtration barrier. Experimentally increasing mTORC1 activity in mouse podocytes induces DN phenotypes, podocyte loss, and mis-localization of Nephrin, a cell surface protein that is important in production of the renal filtration barrier [202], while reduced mTORC1 activity prevents DN progression [202]. Rapamycin and everolimus treatment has also shown therapeutic benefit for DN in other models, including rats with STZ-induced diabetes [203,204,205,206,207]. Some caution is required, however, as mTORC1 activity appears to protect diabetic livers from steatosis [208], though active mTORC2 promotes steatosis through induction of fatty acid and lipid synthesis [209], hence any treatment with mTORC inhibitors in diabetic patients must include close monitoring of a number of biomarkers for liver and kidney function as well as glucose homeostasis.

### 2.8. Age-Related Cancer

Consistent with its role as a central regulator of cell growth, proliferation, and angiogenesis, many oncogenic mutations activate mTOR signalling [210], meaning that the pathway is a key target in anti-cancer therapy. Elderly patients are particularly vulnerable to tumorigenesis; their inflamed tissue microenvironment and the paracrine pro-tumorigenic signalling in the SASP of accumulating senescent cells can drive progression of age-related cancer. In parallel, DNA-damaging chemotherapies given to cancer patients of any age can induce senescence (and the resulting SASP) in both cancerous and healthy collateral cells. This is thought to underlie the increased occurrence of secondary tumours as a side effect of chemotherapy [11,211,212]. Since the SASP is under the control of the mTOR pathway, treating senescent cells with mTOR inhibitors can suppress the secretion of inflammatory cytokines [74,75]. Notably, rapamycin treatment can prevent the stimulation of prostate tumour growth by senescent fibroblasts in mice [74]. Thus, rapamycin may be useful not only as an anti-cancer treatment but also as a preventative therapeutic against age-related cancers or those arising after genotoxic chemo- or radio-therapy.

Despite promising early findings, mTOR inhibitors have not fulfilled their potential as monotherapies against cancer. Combination regimens of mTOR inhibitors together with current best-in class chemotherapeutics do however show efficacy against a range of cancers. For example, combination treatment with rapamycin and resveratrol may be effective in inducing cell death in bladder cancer cells [213], with resveratrol blocking the Akt activation as induced by rapamycin. Similarly, rapamycin has been shown to enhance mitomycin C-induced apoptosis in peritoneal carcinomatosis [214]. In combination with anti-cancer agents, such as trastuzumab or exemestane, mTOR inhibitors exhibit promising anti-tumour activity, even against aromatase inhibitor-resistant breast tumours [215]. Rapamycin may also be beneficial in combination with radiotherapy treatment, for example inducing a significant decrease in tumour metabolic activity of rectal cancers before surgical resection, as assessed by positron emission tomography (PET)-scanning [216].

Currently (June 2018), 461 clinical trials are listed on Clinicaltrials.gov involving the use of mTOR inhibitors in cancer, in a range of tissue types, including breast, cervix, prostate, ovary, pancreas, lung and colon carcinomas, various sarcomas, and lymphomas, while PubMed lists 601 publications for the search terms “mTOR inhibitor cancer clinical trial”. The reported outcomes are highly variable, with some suggesting markedly better outcomes (e.g., Hodgkin’s lymphoma on mTOR inhibition [217,218]), while others showed no improvement or even faster disease progression. It is likely that the variability represents both the stage and grade of cancer, and mTOR status, which should be assessed by ‘personalised medicine’ prior to the use of mTOR inhibitors in cancer treatment, as not all will be driven by hyperactive mTOR, and even those that are may not be sensitive to rapalogue inhibition (e.g., if mutated in the FKBP12 binding site). For those tumours with activated drug-sensitive mTOR, however, mTOR inhibition can give remarkably good outcomes; with the complete response to therapy being reported in one patient during a Phase I trial of everolimus in combination with pazopanib [219]. Use of specific mTORC2 inhibitors has been suggested as route to overcoming the pro-survival effect of PI3K/PDK1/Akt feedback loops [220], though pan-mTOR inhibitors may be equally valuable in this context. The choice to test any drug in aggressive and treatment-refractory or relapsing tumours would present significant challenges, as the cancers by this stage will be genetically heterogeneous and hard to treat; the use of mTOR inhibitors in many such late-stage/refractory cancer trials may therefore not reveal their true potential. It is possible that earlier intervention with mTOR inhibitors, and in combination therapies, may provide more reliable anti-cancer activity. However, a major goal would instead be prevention. In this context, mTOR inhibitors used to intervene in other age-related disease may, in fact, serve a preventative role in cancer, possibly by blocking the deleterious SASP.

## 3. Perspectives

### 3.1. Balancing Efficacy Against Side Effects

Treating otherwise healthy ageing individuals with mTOR inhibitors to treat or prevent progression of age-related disease is only viable if the treatment does not induce unacceptable or undesirable side effects. The studies of immunosenescence from Mannick et al. [110,111] may provide critical insights into side effect profiles of low-dose mTOR inhibition in ageing humans. These studies showed that everolimus and BEZ235 were generally well tolerated, although with an increased incidence of mouth ulceration. Particularly promising is the finding that the two lowest dose regimens of everolimus (0.5 mg daily or 5 mg weekly [111]) proved both the most effective and the best tolerated, with the fewest overall adverse events per cohort. Hence, using as low dose as possible whilst retaining efficacy is critical in minimising side effects.

High dose rapamycin (~20 ng/mL blood) that is used for immunosuppression after transplant or cancer treatment is associated with deleterious side effects, such as the development of type II diabetes [30], though evidence from experimental models produces conflicting results. For example, two short-term studies in mice found that chronic rapamycin treatment induced deleterious metabolic side effects such as weight gain, glucose intolerance [221], and progression of type II diabetes [222], while a longer study showed that these effects could be transient [182]. The dose of rapamycin used may be of critical importance in determining the side effect profile; far lower doses are required for anti-ageing effects than for cancer treatment or immunosuppression and as doses decrease, so do serious adverse events. Disruption of mTORC2 may be behind the metabolic side effects of rapamycin treatment, since it is widely considered that mTORC2 primarily drives the response to insulin signalling and causes lipid biosynthesis (though note the caveats above concerning Akt^S473^ phosphorylation as a sole readout of mTORC2 activity). Carefully considered intermittent treatment regimens may minimize the undesirable effects of rapamycin treatment, such as impaired glucose tolerance [223]. A further alternative strategy to circumvent high dose rapalogue-induced glucose intolerance is to use mTOR inhibitors in combination with anti-diabetes medicines, such as metformin—another promising longevity therapeutic in its own right. Indeed, this strategy has been shown to be highly effective in HET3 female mice treated with both rapamycin and metformin, where glucose tolerance readings were indistinguishable from control mice, though the protective effect was not seen in males [224]. Hence complex-specific mTORC inhibitors, with additional agents to counteract adverse side effects, could retain treatment efficacy over the long-term, a necessary requirement for anti-ageing medicines.

An alternative approach to minimising side effects would be to use a topical application of mTOR inhibitors. This is possible in age-related diseases that occur in discrete compartments, such as OA and AMD, where injection into the affected site is possible. However, as ageing affects the entire body, systemic therapies should be more effective at treating aging per se, and hence in minimising the onset of multiple age-related diseases. mTOR inhibitors currently provide a promising avenue for further research and development, and may promote healthy ageing by modulating the harmful aspects of senescent cells, but they should be considered in combination with other treatment approaches.

In this context, alternative anti-ageing therapies are also being developed—notably the growing field of senolytic drugs that are designed to selectively target and kill senescent cells. These agents exploit the reliance of senescent cells on survival pathways, and they can induce apoptosis specifically in senescent cells, for example, by inducing p53 or disrupting Bcl2. Treatment of aged mice with senolytic agents has been shown to rejuvenate tissues and reverse several age-related pathologies (e.g., [225,226]) and a human clinical trial for senolytic treatment of OA is currently recruiting (Clinicaltrials.gov identifier NCT03513016). However, while senolytics are indisputably exciting, it is well established that senescent cells are beneficial in various instances, such as in wound healing and regeneration. Furthermore, a recent study investigating the senescent cell burden of several tissues of old mice found that up to 14% of cells were senescent [13], with estimates of 20–60% senescent cells in aged primate skin [14,227]. It is therefore important to investigate whether killing a significant proportion of cells in the tissues of elderly patients is safe, whether stem cells are able to refill this empty niche to restore structural and functional tissue integrity, and to assess whether wound healing and regeneration are compromised by senolytic agents. Furthermore, senescent cells from different tissues and in different contexts rely on different survival pathways to avoid apoptosis and are therefore only vulnerable to specific senolytic agents, meaning that a range of senolytics will be required to treat different ARDs. Modulation of the antagonistically pleiotropic and highly heterogeneous state that is cell senescence undoubtedly requires careful and context-dependent consideration.

### 3.2. Monitoring Therapeutic Outcomes: The Need for Ageing Biomarkers

There is an urgent need for reliable, non-invasive, and quantitative biomarkers of senescence and ageing to both measure disease susceptibility or progression, and promptly monitor the outcome of any intervention. It is highly likely that single factors will not be able to adequately reflect the panoply of changes that is associated with ageing and that instead a panel of biomarkers will be required to account for the multi-factorial and complex nature of pathological ageing. Molecular markers that are currently in use include telomere length analysis, DNA methylation patterns, and SAβGAL staining, while functional and morphological markers are also available. The choice of marker may depend on the trial to be conducted—for example, PET scanning for amyloid deposition may be necessary in AD trials, though a recently described blood test for amyloid could substitute [228]. Notably, a number of simple biochemical biomarkers (e.g., glycated haemoglobin) that are selected for inclusion in UK Biobank appear to be valid for assessment of age-related changes, while functional readouts including hand grip strength produce reliable measures of frailty. Clinical trials and any licensed treatments may thus require the development and validation of a panel of biomarkers that could be analysed in a low cost, straightforward, and quick in-house procedure from readily available patient material e.g., urine or blood.

In conclusion, ageing and age-related diseases that arise from hyperactive mTORC signalling may benefit from the use of mTORC inhibitors. However, any such treatment strategy must consider both of the beneficial effects, such as those that are afforded by activation of autophagy and improved quality control of protein synthesis, as well as potential detrimental effects from modifying cellular or organismal metabolism. We believe that mTORC inhibitors hold much promise in the field of anti-ageing medicine, and that clinical prejudice against their use needs to be overcome by careful dosage trials. To obtain maximal therapeutic benefit, whilst minimising side-effects, combinatorial therapies may prove useful. Overall outcomes on ageing and age-related diseases require the use of a panel of robust biomarkers that should provide rapid readouts of age-associated factors in a minimally invasive and cost-effective format. Biochemical pathways that intersect with mTORC signalling may also provide fruitful avenues for anti-ageing drug discovery.

## Figures and Tables

**Table 1:** mTOR complex subunits.

Contribution to Complex	mTORC1	mTORC2
core	mTOR	mTOR
	mLST8/Gβ3	mLST8/Gβ3
	Deptor	Deptor
	Tti1/Tel2	Tti1/Tel2
complex-specific	Raptor	Rictor
	PRAS40	
		mSIN1
		Protor1/2

**Table 2:** Activities and localization of mTORC1 and mTORC2. Note that only a small subset of targets and modulators is shown. Proteins are named using standard nomenclature; for full gene names, please refer to the list of abbreviations.

	mTORC1	mTORC2
localization when active	lysosome	ribosome, plasma membrane, mitochondria, endoplasmic reticulum, lysosome
targets activated	S6K^T389^, HIF 1α, GSK3, SOD1, Grb10, eIF4G, Acinus L, eEF2, IMP2	SGK1, PKC, paxillin, Rho GTPases, Akt^S473^, IGFR, PDK1
targets inhibited	4EBP1/2, Maf1, Lipin-1, ULK1, ATG13, TFEB, DAP1, LARP1	FBW8
activated by	insulin, growth factors, Rheb, Rag, Akt, amino acids, high O_2_, cytokines, TNFα, IkkB	PI3K, growth factors including IGFR, Akt (on mSIN1), membrane tension, ROS, ATM/ATR
inhibited by	AMPK, TSC1/2 (via Rheb inactivation), low O_2_, low ATP, low amino acids	S6K on both Rictor and mSIN1 TSC1/2 (via Rheb inactivation)
biochemical outcomes of activation	protein, nucleotide, lipid and mitochondrial biosynthesis; inhibition of autophagy	actin reorganization, lipid biosynthesis
overall outcomes of activation	cell growth (increase in volume and biomass) cell proliferation suppression of oxidative damage	cell size (surface area increase) cell shape (cytoskeletal changes) survival under oxidative stresscell cycle progression metabolic control

**Table 3:** Classes of mTOR pathway modulators with examples of each class.

Drug Class	Mode of Action	Drug Name	K_i_ or IC_50_	Status
mTORC1 inhibitor	Binds FKBP12 which then associates with mTORC1 and partially occludes kinase active site; mTORC2 inhibited on chronic treatment (possibly through feedback loops)	Rapamycin (sirolimus)	mTORC1 IC_50_ 0.1 nM (in HEK293 cells)	FDA-approved for cancer and as immunosuppressant to prevent rejection in renal transplant; eluting stents in cardiovascular disease Delays senescence in cell culture [109]; extends lifespan and health in lab animals and improves cardiovascular health in companion dogs (see text)
		Everolimus (RAD001)	mTORC1 IC_50_ 1.6–2.4 nM (cell-free assay)	FDA-approved for cancer (e.g., monotherapy against advanced renal cell carcinoma, neuroendocrine tumours of pancreatic, gastrointestinal or lung origin, and SEGA associated with TSC, and as combination therapy with exemestane for HER2-negative breast cancer). Clinical trials show immune system rejuvenation [110,111]
		Temsirolimus; (CCI-779, NSC 683864)	IC_50_ 0.3–0.5 nM in cell culture	FDA approved, used at 10 mg/kg/day in acute lymphocytic leukaemia
Pan-mTOR inhibitor (inhibits both mTORC1 and mTORC2)	ATP-competitive mTORC1/2 inhibitor	AZD8055	mTOR IC_50_ 0.8 nM (MDA-MB-468 cells); 1000-fold selectivity against PI3K isoforms and ATM/DNA-PK	Acceptable safety profile for treatment of advanced solid tumours and lymphoma in phase I trial [112]; reverses phenotypes of senescence in cell culture [34]
		Sapanisertib (AK-228, INK 128, MLN0128)	mTORC1 and mTORC2 1 nM (PI3K isoforms ~200 nM)	Phase 1 trials (cancer)
		OSI-027	22 nM mTORC1, 65 nM mTORC2 (>100× selectivity over PI3K)	Phase 1 trials; in experimental colorectal xenograft, OSI-027 (65 mg/kg) more effective than rapamycin [113], reviewed [114]
mTORC2-specific inhibitor	Prevents interaction of Rictor with mTOR hence blocking mTORC2	JR-AB2		Experimental, xenograft tumour models [115]
Dual PI3K and mTOR inhibitor	ATP-competitive dual PI3K and mTORC1/2 inhibitor	Apitolisib (GDC-0980, RG7422)	Dual PI3K/mTOR 5–14 nM Ki, 17 nM mTOR	Phase 2 trials (cancer)
		Dactolisib (NVP-BEZ235, BEZ235)	mTOR IC_50_ 6 nM, PI3K p110α/γ/δ IC_50_ 4/5/7 nM respectively; IC_50_ ATR 21 nM (cell-free assays)	Passed phase I initial dose discovery trial [116]; modest efficacy in advanced or metastatic carcinoma in phase II [117] but poorly tolerated in advanced pancreatic neuroendocrine tumour patient phase II study [118]; beneficial outcomes in trial with everolimus for reversal of immune senescence [110]
		PF-04691502	PI3K(α/β/δ/γ)/mTOR dual inhibitor with K_i_ of 1.8/2.1/1.6/1.9 and 16 nM (respectively)	Phase 1 clinical trials
PI3K, DNAPK and mTOR	ATP binding site competitor	PI-103	PI3K 2–15 nM, mTOR and DNAPK 30 nM	Experimental [119]
Other components of signalling pathway	PI3K and BRD bromodomain proteins	SF2523	DNAPK 9, 34–158 nM; BRD4 241 nM, mTOR 280 nM	Blocks Brd4; blocks Brd2 to overcome insulin resistance—may be useful as adjunct to prevent diabetic complications of mTOR inhibitors [120]
	Highly selective GSK3 inhibitor; ATP binding competitor	CHIR-98014	GSK3α 0.65 nM GSK3β 0.58 nM	Experimental [121,122]
mTOR activator	FKBP1A	3BDO	N/A	Experimental; inhibits autophagy; provides vascular protection [123]; improves neuronal function in App and Psen1 transgenic mice [124]

IC_50_ and K_i_ data derived from [125].

## Abbreviations

4EBP1	eIF4E binding protein
53BP1	p53 binding protein 1
Aβ	amyloid beta
AD	Alzheimer’s disease
ADPKD	adult polycystic kidney disease
Akt/PKB	protein kinase B
AMD	age-related macular degeneration
AMPK	AMP-activated protein kinase
ARD	age-related disease
ATG13	autophagy related protein 13
ATM	ataxia telangiectasia mutated
ATR	ATM-related
ATP	adenosine triphosphate
CMV	cytomegalovirus
CpG	5’-C-p-G-3’
CR	caloric restriction
DAP1	death associated protein 1
DN	diabetic neuropathy
eEF2	eukaryotic elongation factor 2
eIF	eukaryotic translation initiation factor
ER	endoplasmic reticulum
FA	fatty acid
FBW8	F-Box And WD Repeat Domain Containing 8
FDA	Food and Drug Administration
FKBP	FK506 binding protein
FK506	Tacrolimus
FRB	FKBP12-Rapamycin Binding (FRB) domain of mTOR
GIT1	GPCR-kinase interacting protein 1
GSK3	glycogen synthase kinase 3
HD	Huntington’s disease
HGPS	Hutchinson Gilford progeroid syndrome
HIF1	hypoxia inducible factor 1
HTT	huntingtin protein
IC_50_	half maximal inhibitory concentration
IKK	IkB kinase
IL	Interleukin
IGFR	insulin-like growth factor receptor
IMP2	insulin-like growth factor 2 mRNA binding protein 2
IRS	insulin receptor substrate
Ki	inhibitory constant
LAMTOR	late endosomal/lysosomal adaptor and MAPK and MTOR activator
LAP	liver-enriched activator protein
LARP1	La-related protein
LIP	liver-enriched inhibitory protein
LKB1	liver kinase B1
LMNA	lamin A
L-DOPA	L-dopamine
MAPKAPK2	mitogen-activated protein kinase-activated protein kinase 2
MCF-7	Michigan Cancer Foundation-7 (breast cancer cell line)
mLST8	mammalian lethal with SEC13 protein 8
MMP	matrix metalloproteinase
mTOR	mammalian/mechanistic target of rapamycin
mTORC1/2	mTOR complex 1 or 2
NFAT5	nuclear factor of activated T cells 5
OA	osteoarthritis
PD	Parkinson’s disease
PD-1	programmed death 1
PDK1/2	pyruvate dehydrogenase kinase 1/2
PGC-1-β	peroxisome proliferator-activated receptor gamma coactivator 1-β
PKC	protein kinase C
PPAR	Peroxisome Proliferator Activated Receptor
RA	rheumatoid arthritis
RAD	Ras associated with diabetes
RAGE	receptor for advanced glycation end products
REDD1	regulated in development and DNA damage 1
RNAi	RNA interference
ROS	reactive oxygen species
S6K	protein kinase that phosphorylates S6 ribosomal protein
SAβGAL	senescence associated beta galactosidase
SASP	senescence-associated secretory phenotype
SGK1	serine/threonine protein kinase
SIRT	sirtuin
SOD1	superoxide dismutase 1
SREBP	sterol regulatory element-binding protein
STAT3	signal transducer and activator of transcription 3
T1DMT2DM	type 1 diabetes mellitustype 2 diabetes mellitus
TFEB	transcription factor EB
TNFα	tumour necrosis factor α
TSC1/2	tuberous sclerosis complex 1 or 2
ULK1	Unc-51 like autophagy activating kinase
UTR	untranslated region
VEGF	vascular endothelial growth factor
γH2AX	Ser-139 phosphorylated histone 2A variant X
